# Reference Gene Selection for qPCR Analysis in Tomato-Bipartite Begomovirus Interaction and Validation in Additional Tomato-Virus Pathosystems

**DOI:** 10.1371/journal.pone.0136820

**Published:** 2015-08-28

**Authors:** Ana L. M. Lacerda, Leonardo N. Fonseca, Rosana Blawid, Leonardo S. Boiteux, Simone G. Ribeiro, Ana C. M. Brasileiro

**Affiliations:** 1 Embrapa Recursos Genéticos e Biotecnologia, Embrapa, Brasília, DF, Brazil; 2 Embrapa Hortaliças, Embrapa, Brasília, DF, Brazil; Institute for Sustainable Plant Protection, C.N.R., ITALY

## Abstract

Quantitative Polymerase Chain Reaction (qPCR) is currently the most sensitive technique used for absolute and relative quantification of a target gene transcript, requiring the use of appropriated reference genes for data normalization. To accurately estimate the relative expression of target tomato (*Solanum lycopersicum* L.) genes responsive to several virus species in reverse transcription qPCR analysis, the identification of reliable reference genes is mandatory. In the present study, ten reference genes were analyzed across a set of eight samples: two tomato contrasting genotypes (‘Santa Clara’, susceptible, and its near-isogenic line ‘LAM 157’, resistant); subjected to two treatments (inoculation with Tomato chlorotic mottle virus (ToCMoV) and its mock-inoculated control) and in two distinct times after inoculation (early and late). Reference genes stability was estimated by three statistical programs (geNorm, NormFinder and BestKeeper). To validate the results over broader experimental conditions, a set of ten samples, corresponding to additional three tomato-virus pathosystems that included tospovirus, crinivirus and tymovirus + tobamovirus, was analyzed together with the tomato-ToCMoV pathosystem dataset, using the same algorithms. Taking into account the combined analyses of the ranking order outputs from the three algorithms, *TIP41* and *EF1* were identified as the most stable genes for tomato-ToCMoV pathosystem, and *TIP41* and *EXP* for the four pathosystems together, and selected to be used as reference in the forthcoming expression qPCR analysis of target genes in experimental conditions involving the aforementioned tomato-virus pathosystems.

## Introduction

Tomato (*Solanum lycopersicum* L.) belongs to the Solanaceae family and is one of the most consumed and economically important vegetable crops in the world [1]. Major constraints to tomato production in tropical and subtropical areas are diseases caused by viruses belonging to different genera, such as *Tospovirus*, *Tobamovirus*, *Crinivirus*, *Tymovirus*, and *Begomovirus*. In Brazil, the incidence of diseases in tomato caused by whitefly-transmitted begomoviruses (*Geminiviridae* family) has drastically increased over the recent years with reported yield losses ranging from 40 to 100% [2, 3]. Tomato chlorotic mottle virus (ToCMoV) is a dominant species of the begomovirus complex reported infecting tomatoes in Brazil, having a wide geographic distribution in many important tomato producing areas [2, 4, 5]. Research towards understanding the expression behavior of candidate genes underlying molecular mechanisms of host resistance/susceptibility to viruses involves the use of accurate methods for analyzing gene expression.

Quantitative Polymerase Chain Reaction (qPCR) is currently the most sensitive technique used for gene expression studies [6] either to quantify the input copy number of a particular transcript (absolute quantification) or to measure the relative expression of a target gene (relative quantification). The accuracy and reliability of qPCR analyzes rely on the use of a set of appropriate reference genes for the expression profile normalization [7, 8]. Reference genes are internal controls which the expression must be stable across different samples regardless of experimental conditions and have been largely identified for several plant species, including tomato. However, it was observed that the expression of these so called “housekeeping” genes could vary according to the sample species, genotypes, tissues, developmental stages, treatments, or experimental conditions [9, 10]. Thus, the use of reliable reference genes for normalization is imperative for a consistent analysis of qPCR data, since it eliminates non-biological or methodological-induced variations, allowing a more precise comparison between different mRNA samples [7].

In the recent years, evaluation of reference candidate genes for qPCR analysis in tomato plants has been conducted to identify genes with the most stable expression under given experimental conditions. During tomato development process, four reference genes were chosen as the most stable among 13 candidates [11]. Recently, Dekkers et al. [12] selected nine reference genes expressed in tomato seeds from 24 putative candidates previously identified in *Arabidopsis* microarray data. Lovdal and Lillo [13] evaluated the expression of eight putative reference genes under three major abiotic stresses that affected tomato yield (nitrogen starvation, cold and suboptimal light). On the other hand, for biotic stress, Alfenas-Zerbini et al. [14] identified *APT1* as the most stable reference gene among four candidates during tomato infection by the potyvirus Pepper yellow mosaic virus (PepYMV). Mascia et al. [15] tested the stability of eight reference genes in tomato leaves and roots subjected to the infection of five viruses (including the begomovirus Tomato yellow leaf curl virus; TYLCV) and one viroid and showed thatthe expression of these candidate genes varied to some extent depending on the pathogen and plant tissue analyzed. Those studies together revealed that there is not a “universal” reference gene for tomato and corroborate the consensus concept that several reference genes must be evaluated and more than one selected for given plant genotype, developmental stage and tissue under each experimental condition [10].

One of the main research lines of our team is the study of the bipartite begomovirus-tomato interaction focusing on the role of host genes responsive to infection in contrasting (resistant and susceptible) host plant inbred lines. The identification of reliable reference genes to be used in gene expression analysis by qPCR in our experimental conditions is mandatory. Therefore, the major objective of the present study was to evaluate the stability of ten genes, previously reported as suitable normalizers for qPCR analysis in tomato, aiming to identify a group of reference genes with the most stable expression in-tomato-ToCMoV interaction. For that, the expression of candidate reference genes was analyzed in two contrasting tomato genotypes, inoculated and non inoculated with ToCMoV, and analyzed in two distinct times after inoculation (early and late). Three freely available algorithms–geNorm [16], NormFinder [17] and BestKeeper [18]–were used to identify the two most stable reference genes (*TIP41* and *EF1*) based upon combined analyses of the ranking order outputs from the algorithms. Further validation of the ten candidate reference genes over broader experimental conditions was conducted considering three additional tomato-virus pathosystems besides tomato-ToCMoV: (i) Tomato-GRSV (Groundnut ringspot virus); (ii) Tomato-ToCV (Tomato chlorosis virus); (iii) Tomato-ToBMV (Tomato blistering mosaic virus) + TMV (Tobacco mosaic virus). The results demonstrated that *TIP41* was again rated as the most stable reference gene and, together with *EXP*, could be used for optimal normalization of target tomato genes under a large range of experimental conditions. These reference genes will be valuable tools to optimize the normalization of qPCR data from a wide array of target genes and will be of helpfulness for research groups working in tomato-virus interactions.

## Materials and Methods

### Plant material inoculation

‘Santa Clara’; ‘Moneymaker’ and ‘LAM 144S’ were used in this study as virus susceptible tomato genotypes and ‘LAM 157’ and ‘LAM 144R’ as contrasting virus resistant near-isogenic lines (NILs). ‘LAM 157” harbors the recessive *tcm*-1 locus that confers wide spectrum resistance to bipartite [19] and monopartite [20] begomoviruses. ‘LAM 144R’ carries the dominant *Ty-1* locus that confers resistance several begomoviruses [21].

‘Santa Clara’, ‘LAM 157’, ‘LAM 144S’ and ‘LAM 144R’ plants were inoculated by biolistics with ToCMoV infectious clone as described [5]. Mock-inoculated plants used as control were bombarded with empty pBluescript vector (Agilent Technologies, Inc. Santa Clara, CA, USA). Leaves from each bombarded ‘Santa Clara’ and ‘LAM 157’ plant were collected at 3, 6, 9, 12, and 15 days after inoculation (DAI). Leaves from ‘LAM 144S’ and LAM 144R’ were collected at 25 DAI.

The crinivirus ToCV was transmitted by whiteflies and the tospovirus GRSV was mechanically inoculated into ‘Santa Clara’ plants. Inoculated and non inoculated control leaves were collected 100 DAI. ‘Moneymaker’ plants were mechanically inoculated with a mixture of the tymovirus ToBMV and the tobamovirus TMV. Inoculated and non inoculated control leaves were collected at 18 DAI.

All collected leaves were frozen in liquid nitrogen immediately after harvesting, and total RNA was extracted using the RNeasy Plant Mini Kit (Qiagen, Valencia, CA, USA).

### RNA sampling and cDNA synthesis

For ‘Santa Clara’ and ‘LAM 157’inoculated with ToCMoV, RNA samples from four bombarded plants were combined in equal quantities per collecting point (3, 6, 9, 12, and 15 DAI) and for each treatment (ToCMoV-inoculated and mock-control). Four sample pools for each genotype were formed afterward by combining equal amounts of total RNA from 3 and 6 (early) and 9, 12 and 15 (late) DAI in each treatment.

For the other pathosystems, RNA from ten samples was considered: (i) ‘LAM 144S’-ToCMoV; (ii) ‘LAM 144R’-ToCMoV; (iii) ‘Santa Clara’-ToCV; (iv) ‘Santa Clara’- GRSV;(v) ‘Moneymaker’-ToBMV+TMV and their respective non inoculated controls.

RNA integrity inspection, DNAse treatment, and cDNA synthesis were carried out, as previously described [22].

### Primer design and qPCR reaction

Ten candidate reference genes were selected to identify the most stably expressed to be used for qPCR analysis in our experimental conditions (Table 1). This set of genes comprised nine “classic” reference genes which have been previously assigned as appropriate internal control for qPCR studies in tomato [11, 14, 15]. An additional candidate gene, *Phytoene desaturase* (*PDS*), was also selected since it displayed a very stable expression in previous qPCR analysis conducted so far in tomato in our laboratory (data not shown). Primers for the reference gene *PDS* and the two target genes here analyzed [*Necrotic spotted lesions 1* gene (*NECRO*) and *Nicotiana lesion-inducing like—HR-like lesion-inducer* gene (*HR-Li*)] were designed with Primer3Plus software [23] (Table 1). The following parameters were used: melting temperature between 55–62°C, primer lengths 19–22 bp and amplicon length between 150 and 200 bp.

**Table 1:** Description of the reference and target genes used for qPCR analysis.

Gene name	Gene Symbol	Accession number^a^	Primer sequence (5’-3’)	Amplicon size (pb)	Amplification efficiency ± SD^b^	Reference
**Actin**	*ACT*	BT013524	CGGTGACCACTTTCCGATCT	62	1.038 ± 0.0077	[14]
			TCCTCACCGTCAGCCATTTT			
**β-6 Tubulin**	*TUB*	BT013153	TTGGTTTTGCACCACTGACTTC	84	0.987 ± 0.0070	[14]
			AAGCTCTGGCACTGTCAAAGC			
**Adenine phosphoribosiltransferase 1**	*APT*	BT012816	GAACAGACAAGATTGAGATGCATGTA	60	0.999 ± 0.0072	[14]
			CCACGAGGGCACGTTCA			
**Phytoene Desaturase**	*PDS*	NM_001247166	GCCGATTGTGGAACATATTGAGTC	91	1.027 ± 0.0085	Our library
			GACACTTCCATCCTCATTCAGCTC			
**Expressed protein**	*EXP*	SGN-U346908	GCTAAGAACGCTGGACCTAATG	183	1.052 ± 0.0110	[11]
			TGGGTGTGCCTTTCTGAATG			
**TIP 41 (TAP42-interacting protein)**	*TIP41*	SGN-U584254	ATGGAGTTTTTGAGTCTTCTGC	235	1.018 ± 0.0067	[11]
			GCTGCGTTTCTGGCTTAGG			
**Clathrin adaptor complexes medium subunit**	*CAC*	SGN-U314153	CCTCCGTTGTGATGTAACTGG	173	1.107 ± 0.0184	[11]
			ATTGGTGGAAAGTAACATCATCG			
**Glyceraldehyde 3-phosphate dehydrogenase**	*GAPDH*	U93208	ACCACAAATTGCCTTGCTCCCTTG	110	1.012 ± 0.0099	[15]
			ATCAACGGTCTTCTGAGTGGCTGT			
**Ubiquitin 3**	*UBI*	X58253	TCGTAAGGAGTGCCCTAATGCTGA	119	0.956 ± 0.034	[15]
			CAATCGCCTCCAGCCTTGTTGTAA			
**Elongation factor 1α**	*EF1*	BT013246	GATTGACAGACGTTCTGGTAAGGA	67	1.040 ± 0.0089	[14]
			ACCGGCATCACCATTCTTCA			
**Necrotic spotted lesions 1 gene**	*NECRO*	SGN-U581658	AACCTGTTTGCCGGCATCTCCA	193	0.975±0,0062	Our library
			GTGTCCCAACATGGCAGAAGGA			
**Nicotiana lesion-inducing like—HR-like lesion-inducer gene**	*HR-Li*	SGN-U581658	ATTGGCTCCCAAAGTGGCTGGT	154	1.043±0.0117	Our library
			AGCACCAGTCATGCTGCCAA			

^a^Accession numbers from GenBank database or Sol Genomics Network (SGN).

^b^Amplification efficiency was calculated using the online real-time PCR Miner tool [25].

qPCR reactions were carried out in 96-well plates on a 7300 Real-Time PCR System (Applied Biosystems, Foster City, CA, USA) as previously described [24]. Reactions were conducted in three technical replicates for each sample. No template control (NTC) and no amplification control (NAC) using total RNA as template were included as negative controls for each master mix.

### Stability analysis of reference genes

PCR amplification efficiency and optimal cycle of quantification (Cq) values of all samples were calculated from raw fluorescence data using the online real-time PCR Miner tool [25], as previously described [24]. These data were used to assess reference genes stability by three statistical programs: geNorm^PLUS^ [16], NormFinder version 0.953 [17] and BestKeeper version 1 [18].

For ‘Santa Clara’ and ‘LAM 157’inoculated with ToCMoV, the stability data of each program for ten reference genes were analyzed considering either all samples together (entire dataset) or in six separate subsets: ‘Santa Clara’ and ‘LAM 157’ (genotype subsets); early and late (time after inoculation subsets) and inoculated and control (treatment subsets). For the additional pathosystems, stability data of each program for ten reference genes were analyzed considering all ten new samples together with the eight former samples from ‘Santa Clara’ and ‘LAM 157’inoculated with ToCMoV, resulting in an entire dataset of 18 samples.

To validate the use of the reference genes in all experimental conditions studied, the expression profile of two target genes (*NECRO* and *HR-Li*) was normalized using the two most stable candidate reference genes and the two least stable, as determined by the combined ranking order outputs from geNorm, NormFinder and BestKeeper algorithms. The relative expression of *NECRO* and *HR-Li* target genes after virus infection in all pathosystems here studied was estimated and statistically tested using REST 2009 software version 2.0.13 [26], as previously described [24].

## Results

### Expression profiles of candidate reference genes

**Fig 1:**
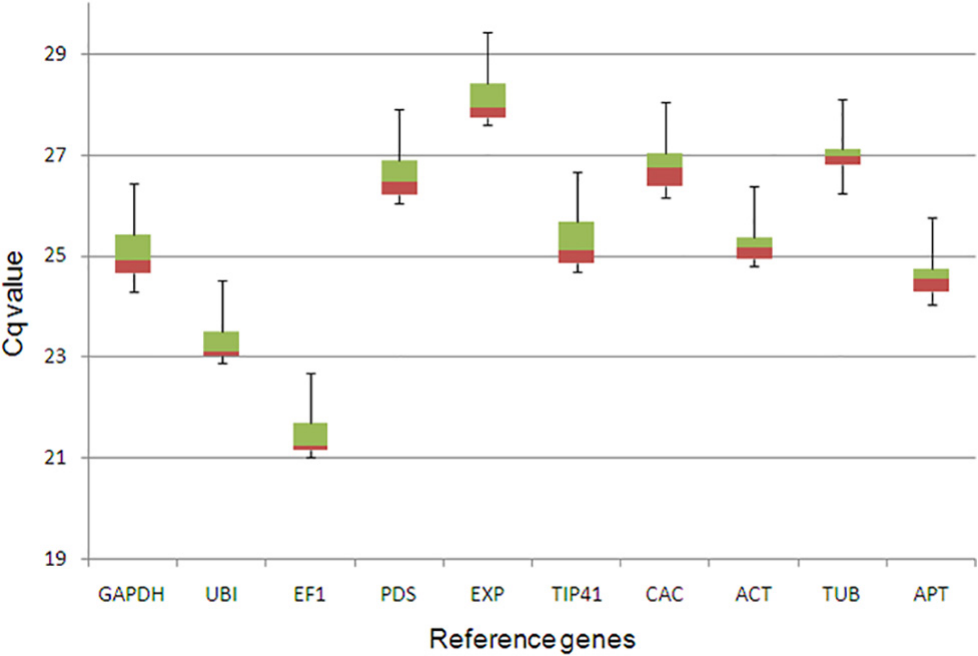
Cycle of quantification (Cq) values distribution of candidate reference genes. Box-whisker plot showing Cq values distribution of each reference gene considering all samples together (entire dataset). The quartiles with medians are represented by boxes green (upper) and red (lower). Whiskers represent the maximum and minimum values.

To properly achieve our expression studies on the tomato-ToCMoV pathosystem, ten candidate reference tomato genes were selected (Table 1), and their expression stability assigned in inoculated and non-inoculated ‘Santa Clara’ (susceptible) and ‘LAM 157’ (resistant) tomato genotypes. Following qPCR reactions, raw fluorescence data was directly used by the Miner statistical algorithm [25] for Cq and primer efficiency calculations (Table 1). This tool has proven to be accurate, simple and user-friendly as it does not require the establishment of a standard curve, as previously observed [24]. All tested primer pairs showed very high efficiency, ranging from 0.95 to 1.10 (Table 1) and were used to adjust Cq values in subsequent qPCR analysis. The expression level of the ten candidate reference genes, presented as Cq values, varied between 21 and 28, with most of them lying between 24 and 27 (Fig 1). *EF1* was the most highly expressed (with a mean Cq of 21.41), and *EXP* the least expressed (mean Cq of 28.09). *TUB* showed the least variation (CV of 0.33%) while *TIP41* (CV of 0.48%) was the most variable (Fig 1). The melt curve analysis confirmed the amplification specificity of all transcripts, as a single peak was generated. No primer dimers formation was observed. No amplification was detected in the absence of template (NTC control) or when RNA was used as template (NAC control). In order to choose the best reference genes, three programs (geNorm, NormFinder, and Bestkeeper) were employed aiming to obtain a more comprehensive assessment of the gene expression data.

### Analysis using the geNorm algorithm

The algorithm geNorm [16] determines the variation level in the expression (M) of each reference gene compared with the expression of all other genes under the studied experimental conditions. The best reference gene is the one that has the most similar levels of expression in all samples regardless of the experimental condition.

The average expression M values calculated by geNorm for the ten reference genes here tested are presented in Table 2. Considering the entire dataset, the two most stable reference genes were *EXP* and *TIP41*, with the lowest M values (M = 0.135 and 0.143, respectively). On the other hand, the M values of *TUB* and *ACT* (M = 0.273 and 0.254, respectively) were the highest, indicating that those genes were most variably expressed. However, the results changed when the data was analyzed by subsets (S1 Fig). Analyses using the genotype subsets pointed out *EF1* and *UBI* (M = 0.045 and 0.052, respectively) as the best reference genes for ‘Santa Clara’ whilst *APT* and *GAPDH* were the most stable genes (M = 0.079 and 0.081, respectively) for ‘LAM 157’. For the treatment subsets, in samples inoculated with ToCMoV, *CA*C and *ACT* (M = 0.081 and 0.082, respectively) showed to be the most stable genes while *GAPDH* and *EXP* (M = 0.028 and 0.031, respectively) had the highest expression stability in the corresponding non-inoculated control samples. When considering the time subsets, *EXP* and *CAC* (M = 0.086 and 0.093, respectively) were expressed much more stable than the other reference genes at early time points (3 and 6 DAI) and *TIP41* and *EXP* (M = 0.130 and 0.131, respectively) at late times (9 to 15 DAI).

**Table 2:** Evaluation of expression stability of reference genes by geNorm, NormFinder, and BestKeeper, considering the entire dataset of ‘Santa Clara’- and ‘LAM 157’-ToCMoV samples.

	geNorm		NormFinder		BestKeeper	
Ranking order	Gene Symbol	Stability value (M)	Gene Symbol	Stability Value (M)	Gene Symbol	Corr.Coef. (*r*)
**1**	*EXP*	0.135	*EF1*	0.008	*TIP41*	0.974
**2**	*TIP41*	0.143	*EXP*	0.010	*EF1*	0.972
**3**	*EF1*	0.151	*TIP41*	0.011	*GAPDH*	0.961
**4**	*CAC*	0.167	*CAC*	0.012	*EXP*	0.959
**5**	*GAPDH*	0.182	*UBI*	0.014	*CAC*	0.943
**6**	*UBI*	0.203	*PDS*	0.017	*UBI*	0.894
**7**	*APT*	0.222	*GAPDH*	0.018	*PDS*	0.893
**8**	*PDS*	0.238	*APT*	0.018	*APT*	0.877
**9**	*ACT*	0.254	*ACT*	0.019	*ACT*	0.817
**10**	*TUB*	0.273	*TUB*	0.025	*TUB*	0.675

All genes displayed high stability levels over the entire dataset and the six subsets since all M values are ≤ 0.273, far below the geNorm accepted threshold of 1.5 [16]. This high stability is most likely related to the accurate selection of the reference genes, which were previously validated in other studies [11, 14, 15]. The use of reference genes with highly stable expression increases the accuracy of gene expression analysis by qPCR since it allows a better discrimination of even small differences among the samples.

**Fig 2:**
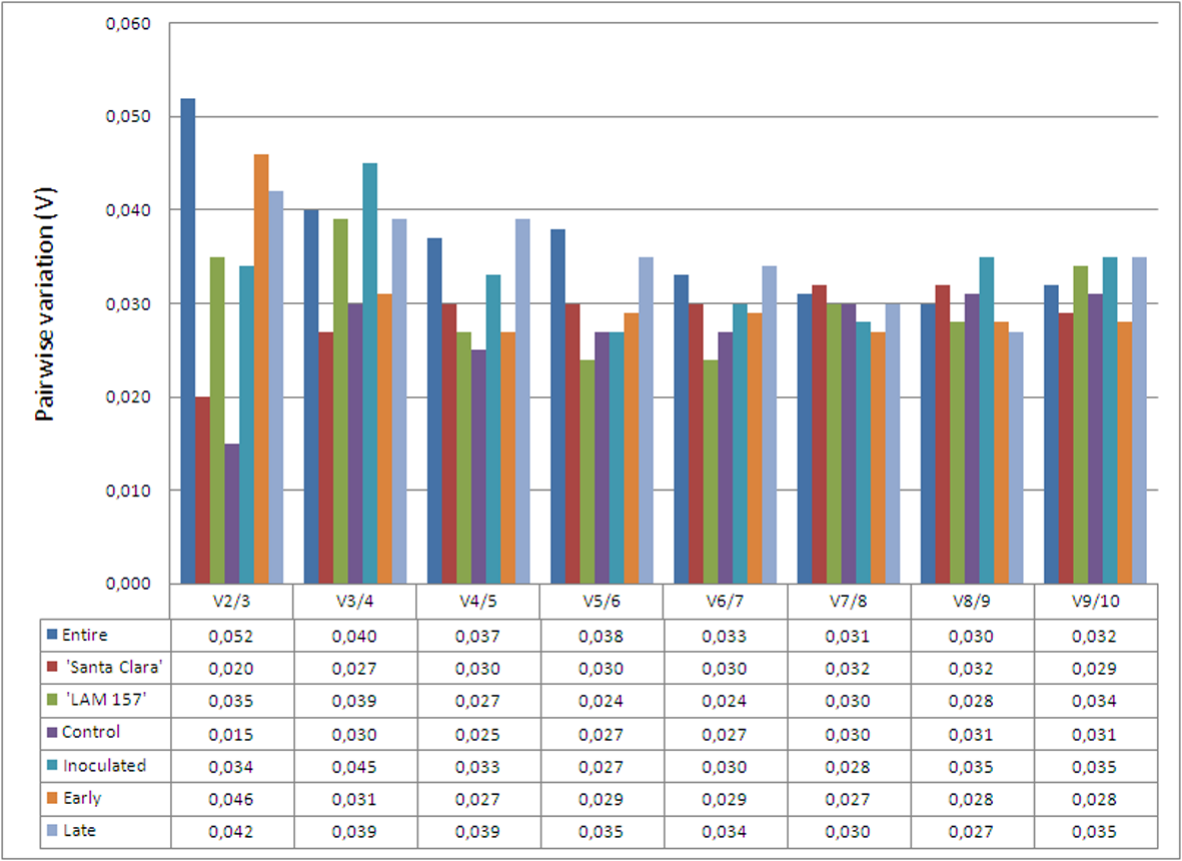
Pairwise variation (V) of candidate reference genes as predicted by geNorm. Pairwise variation (Vn/Vn+1) for determination of the optimal number of reference genes predicted by geNorm and calculated between the normalization factors NFn and NFn+1, with a recommended cutoff threshold of 0.150. The pairwise variation was analyzed considering either all samples together (entire dataset) or in six separate subsets: ‘Santa Clara’ and ‘LAM 157’ (genotype subsets); control and inoculated (treatment subsets) and early and late (time after inoculation subsets).

Pairwise variation (V_n/n+1_) between consecutively ranked NFs was subsequently calculated by geNorm to determine the effect of adding the next reference gene to those already analyzed. Below the recommended cutoff value of 0.150, the inclusion of an additional reference gene will not have a significant effect on the normalization [16]. The analysis indicated that, when all the samples were taken together, the pairwise variation V_2/3_ was 0.052 (Fig 2). This result suggested that for the entire dataset only the two most stable reference genes determined by geNorm, *EXP* and *TIP41*, would be necessary for an optimal normalization of qPCR data. The addition of the third reference gene (*EF1*, in this case) would not improve the reliability of the normalization (Fig 2). The same result was observed for the other subsets. The low value of pairwise variation (all V_n/n+1_ values are ≤ 0.052) reflects the high stability of the genes here analyzed when comparing with other reference genes studies in tomato [11–15]. In fact, low pairwise variation is uncommon but was also observed in the citrus-Citrus leprosis virus C (CiLV-C) pathosystem [27].

### Analysis using the NormFinder algorithm

NormFinder [17] is another tool that calculates the stability index (M) of genes taking into account the variation in gene expression across all samples and experimental conditions (intra- and inter-treatments). NormFinder analysis showed that *EF1*, *EXP*, and *TIP41* had a remarkable stability of their expression levels (Table 2 and S1 Fig). Excepted for inoculated treatment, these reference genes were classified as the top three positions when samples were evaluated across the entire dataset or in separate subsets. On the other hand, *TUB*, *ACT*, and *APT* exhibited unstable expression profiles and were most often included among the least stable reference genes (Table 2 and S1 Fig). As observed in geNorm analysis, M values obtained by NormFinder were also very low (all values are ≤ 0.028), also indicating the high expression stability of candidate genes.

### Analysis using the Bestkeeper algorithm

BestKeeper [18] estimates the average Cq values of the candidate reference genes in all tested conditions and the Pearson correlation coefficient (*r*) to indicate the best reference gene according to gene-correlation analysis of candidate gene pairs. In a positive correlation, more stable gene expression is indicated by higher correlation coefficient *r* values.

When considering the entire dataset, *r* values were high (close to the maximum value of 1.0 admitted by Bestkeeper) for the ten reference genes and indicated *TIP41* and *EF1* as most stable genes with similar *r* values of 0.974 and 0.942, respectively (Table 2). *TUB* and *ACT* ranked as the least stable genes with *r* = 0.675 and 0.817, respectively. Further data processing from six subsets showed a gene ranking that slightly differs from the entire dataset (S1 Fig). Genotype subsets assessed *EXP* and *UBI* as the most stable reference genes for ‘Santa Clara’, and *APT* and *GAPDH* for ‘LAM 157’. For virus-inoculated samples, *APT* and *TIP41* were the most stable genes whereas the analysis of mock-inoculated controls revealed that *CAC*, *UBI*, and *GAPDH* were the most stable ones. For both early and late samples, *TIP41* was the most stable gene. In late subset, the *r* value for *TUB* gene is remarkably low (*r* = 0.168), unlike the other reference genes that showed high stabilities with inconspicuous variation (S1 Fig).

### Ranking order of the most stable genes

The rank of gene expression stability obtained in this study varied with the statistical programs used and the analyzed subsets (Table 3 and Table 4), and a consensus among the results was not possible. These results are consistent with the literature that has suggested that geNorm, NormFinder, and Bestkeeper tools are more likely to generate distinct ranking orders of reference genes since they are based on different algorithms and analytical procedures. They should, therefore, be considered complementary statistical methods. Thus, to select the best reference genes to be used in our experimental conditions, the arithmetic mean ranking value of each gene obtained when combining all the three statistical program outputs was calculated [28]. Table 3 displays gene stability ranking order obtained when the entire dataset was analyzed, taking into consideration the integrated results of the three programs. Genes were ranked in positions 1 to 10 according to their arithmetic mean. *TIP41*, *EF1*, and *EXP* were rated as the most stable, with a very similar ranking mean value (2, 2, and 2.67). *TUB* and *ACT* were ranked at 10^th^ and 9^th^ positions, respectively, in the three statistical program analyses (Table 3) and were, therefore, classified as the least stable under our experimental conditions.

**Table 3:** Gene stability ranking order by geNorm, NormFinder and BestKeeper; and their respective arithmetic mean ranking values, considering the entire dataset of ‘Santa Clara’-ToCMoV and ‘LAM 157’-ToCMoV samples.

Ranking order	Genes	GeNorm	NormFinder	BestKeeper	Mean value
**1**	*TIP41*	2	3	1	2
**2**	*EF1*	3	1	2	2
**3**	*EXP*	2	2	4	2.67
**4**	*CAC*	4	4	5	4.33
**5**	*GAPDH*	5	7	3	5
**6**	*UBI*	6	5	6	5.67
**7**	*PDS*	8	6	7	7
**8**	*APT*	7	8	8	7.67
**9**	*ACT*	9	9	9	9
**10**	*TUB*	10	10	10	10

**Table 4:** Gene stability ranking order obtained by geNorm, NormFinder and BestKeeper, considering six subsets of ‘Santa Clara’ and ‘LAM 157’ samples inoculated with ToCMoV.

Ranking order	Subsets					
	Genotype		Treatment		Time	
	‘Santa Clara’	‘LAM 157’	Control	Inoculated	Early	Late
**1**	*UBI*	*EF1*	*EXP*	*APT*	*TIP41*	*EXP*
**2**	*EXP*	*TIP41*	*GAPDH*	*CAC*	*EXP*	*TIP41*
**3**	*EF1*	*GAPDH*	*CAC*	*ACT*	*EF1*	*PDS*
**4**	*CAC*	*APT*	*EF1*	*TIP41*	*GAPDH*	*EF1*
**5**	*ACT*	*CAC*	*UBI*	*EF1*	*CAC*	*CAC*
**6**	*TIP41*	*UBI*	*APT*	*EXP*	*TUB*	*ACT*
**7**	*PDS*	*EXP*	*PDS*	*GAPDH*	*PDS*	*UBI*
**8**	*GAPDH*	*PDS*	*TIP41*	*UBI*	*UBI*	*GAPDH*
**9**	*TUB*	*TUB*	*ACT*	*PDS*	*APT*	*APT*
**10**	*APT*	*ACT*	*TUB*	*TUB*	*ACT*	*TUB*

As expected, analysis of the best reference genes taking into account each experimental subset showed some differences (Table 4). The three genes sorted as the most stable when the entire dataset was considered, i.e., *TIP41*, *EF1* and *EXP* (Table 3) also appeared at the top four positions for the subsets associated with the elapsed time after virus inoculation (time subsets; Table 4). However, for the other subsets, the ranking of these genes was variable. For ‘Santa Clara’ subset, *UBI* was classified as the most stable followed by *EXP* and *EF1* whereas for ‘LAM 157’, *EF1*, *TIP41* and *GAPDH* were the genes that showed the higher stability. In the treatment subsets, *EXP* and *EF1* were ranked as the first and fourth most stable genes in control whereas, in inoculated samples, their status changed to the sixth and fifth positions, respectively. *TUB* was again ranked as one of the less stable gene, excepted in the early subset (Table 4). Interestingly, *APT* and *ACT*, pointed out as the least stably expressed genes in the majority of subsets, were ranked at first and third position, respectively, in the inoculated subset (Table 4).

In genotype and time subsets, the gene ranking was relatively conserved between samples. However, in the treatment subsets, the rank of genes has not been maintained and changed at least six positions between samples, as for *ACT*, *EXP*, *GAPDH* and *APT* (Table 4).

### Gene stability analysis in additional tomato-virus pathosystems

To validate the reliability of the selected reference genes identified in tomato–ToCMoV pathosystem over broader experimental conditions, a set of new ten samples was further analyzed together with the previous eight samples using the same three algorithms (geNorm, NormFinder, and Bestkeeper). Therefore, this analysis considered as an entire dataset the 18 samples corresponding to the interaction of ‘Santa Clara’ susceptible tomato genotype with three viruses (ToCMoV, ToCV and GRSV) and ‘Moneymaker’, also a susceptible genotype, with a mixture of viruses(ToBMV and TMV) and three ‘LAM’ genotypes (‘LAM 144S’, susceptible, ‘LAM 144R’ and ‘LAM 157’, resistant) with ToCMoV. The stability of the ten candidate reference genes was assessed in this new dataset. As expected, the ranking order of gene stability diverged according to three algorithms used (Table 5). *APT*, *TUB*, and *TIP41* were the most stable when analyzed by geNorm; *TIP41*, *ACT*, and *EXP* by NormFinder and *TIP41*, *EXP*, and *CAC* by Bestkeeper. The sorting of the genes by the arithmetic mean of the ranking values indicated that *TIP41* and *EXP* are the two most stable genes (Table 5). They were followed by *APT* and *CAC* in the third and fourth places, respectively. *EF1* was surprisingly ranked as the least stable gene, in disagreement with previous results obtained with ‘Santa Clara’ and ‘LAM 157’ genotypes inoculated with ToCMoV that ranked this gene as the second most stable Table 3).

**Table 5:** Gene stability ranking order by geNorm, NormFinder and BestKeeper, considering 18 samples of ‘Santa Clara’; ‘LAM 157’; ‘Moneymaker’ and ‘LAM 144’ inoculated with ToCV; GRSV; ToBMV+ TMV or ToCMoV.

Ranking order	Genes	GeNorm	NormFinder	BestKeeper	Mean value
1	*TIP41*	3	1	1	1.6
2	*EXP*	4	3	2	3.0
3	*APT*	1	5	5	3.6
4	*CAC*	6	4	3	4.3
5	*ACT*	7	2	4	4.3
6	*PDS*	5	6	6	5.6
7	*TUB*	2	8	9	6.3
8	*UBI*	8	7	10	8.3
9	*GAPDH*	10	9	7	8.6
10	*EF1*	9	10	8	9

The analysis conducted with all pathosystem samples together also showed very high stability measurements (M or *r* values) for the ten reference genes using the three algorithms (data not shown). This result indicates the high stability of these ten genes regardless of the tomato-virus pathosystem analyzed or the stability ranking order.

### Validation of the selected reference genes

To validate the selection of reference genes, the relative expression level of target genes was analyzed. For data normalization, the two most stable (*TIP41* and *EF1*) and the two least stable genes (*TUB* and *ACT*) were used, in accordance with the results obtained with all tomato-virus pathosystems here analyzed (Tables 3 and 5). Two genes associated with cell death and defense response in plants, *NECRO* and *HR-Li* [29, 30] and potentially involved in host response to begomovirus infection were chosen as target genes (Table 1). In a first series of expression analysis, the differential expression of these target genes was evaluated in ToCMoV-inoculated ‘LAM 157’ plants (resistant genotype) at early (3 and 6 DAI) or late (9, 12 and 15 DAI) times after inoculation relative to mock-inoculated controls.

**Fig 3:**
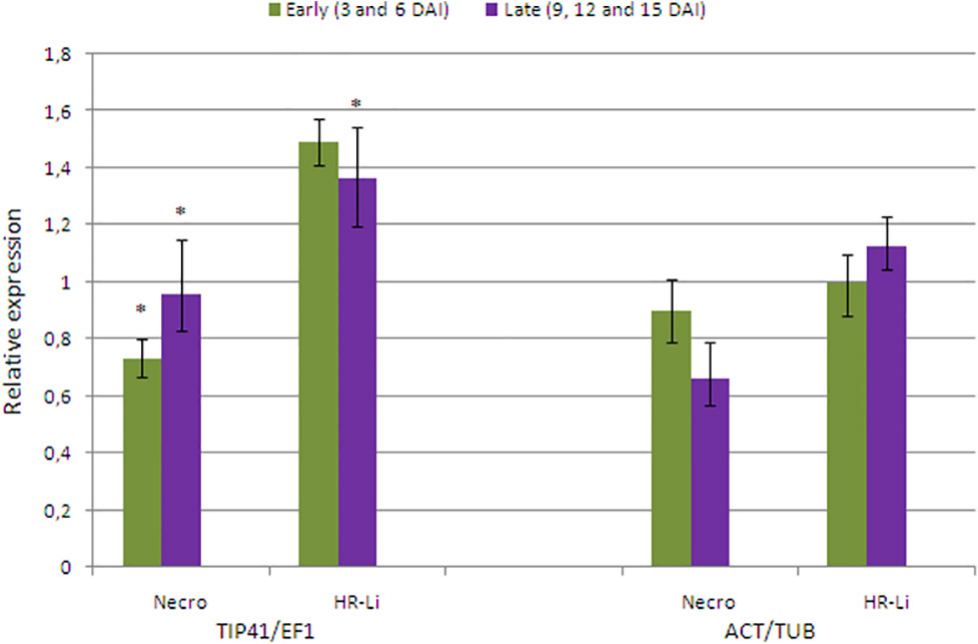
Expression quantification of target genes in ‘LAM 157’-ToCMoV interaction using different combinations of normalizers. The normalization of relative expression data was made with two combinations of reference genes: the two most stable genes (*TIP41* and *EF1*) and the two least stable genes (*ACT* and *TUB*) genes. The relative expression of two target genes [*Necrotic spotted lesions 1* (*NECRO*) and *HR-like lesion inducer* (*HR-Li*)] was evaluated in ToCMoV inoculated plants of ‘LAM 157’ (resistant genotype) at early (3 and 6 DAI) and late (9, 12 and 15 DAI) times after virus inoculation relative to their respective mock-inoculated controls. Error bars represent the standard deviation of three technical replicates.

qPCR analysis showed that the expression of *NECRO* gene was downregulated after ToCMoV inoculation (Fig 3). The level of expression was significantly downregulated in both early (RQ = 0.731) and late (RQ = 0.959) infection times when the expression was normalized with the two most stable reference genes (*TIP41* and *EF1;* Fig 3). Conversely, when the samples were normalized with the two least stable reference genes (*ACT* and *TUB*) the expression pattern changed (Fig 3). The relative expression of *NECRO* gene increased in early infection (RQ = 0.900) while in late infection it decreased (RQ = 0.662; Fig 3).

Likewise, for *HR-Li* gene, the expression pattern changed according to the combination of reference genes used for normalization. At early time, *HR-Li* showed an upregulation in response to ToCMoV inoculation, with higher values when used *TIP41* and *EF1* as reference genes (RQ = 1.462) than with *ACT* and *TUB* (RQ = 1.001; Fig 3). At late time, the same behavior was observed in differential expression of *HR-Li* with a significant higher upregulation when used *TIP41* and *EF1* as reference genes (RQ = 1.362) than with *ACT* and *TUB* (RQ = 1. 128; Fig 3).

**Fig 4:**
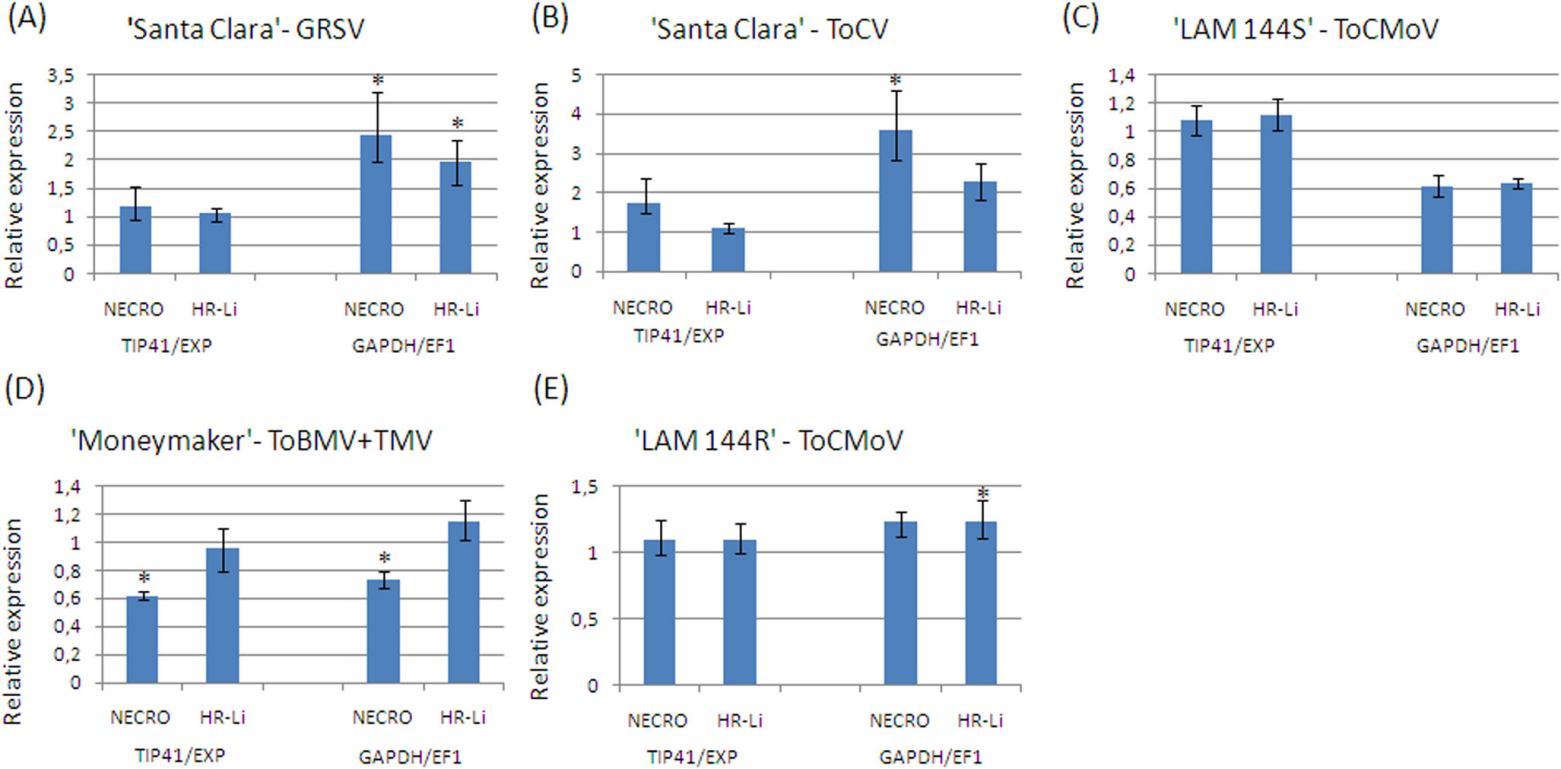
Expression quantification of target genes in four tomato-virus-pathosystems using different combinations of normalizers. The normalization of relative expression data was made with two combinations of reference genes: the two most stable genes (*TIP41* and *EXP*) and the two least stable genes (*GAPDH* and *EF1*) genes. The relative expression of two target genes [*Necrotic spotted lesions 1* (*NECRO*) and *HR-like lesion inducer* (*HR-Li*)] was evaluated in five pathosystems after virus inoculation relative to their respective mock-inoculated controls. (A) ‘Santa Clara’ 100 DAI with GRSV; (B) ‘Santa Clara’ 100 DAI with ToCV; (C) ‘LAM 144S’ 25 DAI with ToCMoV; (D)‘Moneymaker’ 18 DAI with ToBMV and (E) ‘LAM 144R’ 25 DAI with ToCMoV. Error bars represent the standard deviation of three technical replicates.

Additional expression analysis of *NECRO* and *HR-Li* target genes was conducted to validate the reference genes selected when all samples of the four studied pathosystems were considered as experimental conditions. In this analysis, the differential expression of target genes was evaluated in inoculated plants relative to non inoculated control plants. As expected, *NECRO* and *HR-Li* expression profile varies according to the pathosystem studied and the combination of normalizers used (Fig 4).

In the samples of 'Santa Clara' inoculated with GRSV, the relative expression of *NECRO* and *HR-Li* gene changes according to the couple of reference genes used (Fig 4A). The expression of these target genes is significantly upregulated by the virus inoculation when normalized with *GAPDH* and *EF1* (RQ = 2.433 and RQ = 1.972, respectively; Fig 4A). However, when normalized with the most stable genes (*TIP41* and *EXP*), *NECRO* and *HR-Li* relative expressions are also positive but with lower RQ values (1.173 and 1.05 for *NECRO* and *HR-Li* genes, respectively; Fig 4A).

Likewise, in ‘Santa Clara’ plants inoculated with ToCV, the expression behavior of *NECRO* and *HR-Li* genes is distinct regarding the couple of reference genes used (Fig 4B). When *NECRO* expression was normalized with the less stable reference genes (*GAPDH* and *EF1*), its relative expression is higher (RQ = 3.604) and statistically significant than when using the most stable genes (RQ = 1.741). Moreover, *HR-Li* relative expression is higher using *GAPDH* and *EF1* as references (RQ = 2.269) than *TIP41* and *EXP* (RQ = 1.096).

Also being a susceptible genotype, in ‘LAM 144S’ plants the *NECRO* and *HR-LI* expression behavior in response to ToCMoV inoculation is very similar to Santa Clara with GRSV and ToCV and change according the reference genes used (Fig 4C). In these samples, the *NECRO* and *HR-LI* relative expression is almost 1.8 times higher when normalized with *TIP41* and *EXP* (RQ = 1.077 and RQ = 1.118, respectively), compared to *GAPDH* and *EF1* (RQ = 0.615 and RQ = 0.638; (Fig 4C).

On the other hand, relative expression analyses of target genes in ‘Moneymaker’ (Fig 4D) and ‘LAM 144R’ (Fig 4E) genotypes inoculated with ToBMV+TMV and ToCMoV, respectively, showed similar patterns and RQ values when the expression was normalized by *TIP41* and *EXP* or by *GAPDH* and *EF1*.

Collectively these expression analyses indicated that the choice of reference genes could have a critical effect on the normalization of relative expression values of target genes. Thus, our results confirm the concept that it is essential to evaluate the stability of several candidate reference genes and select those with proven expression stability in each experimental system prior to its use in qPCR analysis.

## Discussion

Quantitative PCR (qPCR) is a technique widely used to analyze relative gene expression due to its accuracy and versatility. The adoption of reliable reference genes for data normalization of target gene expression is indispensable to achieve a proper and consistent qPCR analysis and interpretation.

Several works in many plant species such as *Arabidopsis* [31]; cotton [32]; peanut [22]; *Brachiaria* [33]; and soybean [34] have supported the importance of identifying the best combination of more than one reference gene for each species and experimental condition studied. Recent gene expression studies involving interactions between the begomovirus TYLCV and tomato [35, 36] and Pepper golden mosaic virus (PepGMV) and pepper [37] have used *TUB* as reference gene for expression quantification of several target genes putatively involved in the pathosystems.

In the present work, the expression stability of *PDS* and nine other candidates, previously identified as good tomato reference genes during fruit development [11] or under biotic [14, 15] and abiotic [13] stresses, were analyzed. Each mentioned study identified different reference genes as ideal for normalization of qPCR data, evidencing that a prior careful evaluation of reference genes stability is necessary for each experimental setup. Therefore, in a first series of gene stability expression analysis, we have assessed ten reference genes, including “classical” and new candidates, for qPCR normalization during the begomovirus ToCMoV interaction with ‘Santa Clara’ and ‘LAM 157’, which are susceptible and resistant tomato genotypes, respectively. The two genotypes were inoculated with ToCMoV and the stability of candidate reference genes was analyzed considering all samples together (the entire dataset) or in three groups of subsets (genotype, treatment and time subsets).

The three statistical programs used to estimate gene stability, geNorm [16]; NormFinder [17]; and BestKeeper [18] showed divergent classification of most stable genes. The application of these three algorithms delivered identical ranking in only two (*ACT* and *TUB*) out of the investigated ten reference genes (Table 3). Interestingly, those genes are the two least stable. This apparently contradictory result corroborates previous works showing that those programs are complementary and that the use more than two statistical algorithms should be applied to better assess reference gene stability [11, 38].

Additional reference genes stability analysis was further carried out over broader experimental conditions. This analysis included tomato-virus pathosystems involving five tomato genotypes (‘Santa Clara’; ‘LAM 157’; ‘Moneymaker’; ‘LAM 144S’ and ‘LAM 144R’) with four virus combinations (ToCMoV; GRSV; ToCV;, and ToBMV+TMV). Besides the pathosystems, this set of samples also considered differently aged leaves (from 3 to 100 DAI) and three inoculation methods (biolistics; whitefly and mechanical transmissions). The stability of the ten candidate reference genes was assessed and few differences were observed in the gene ranking when compared to the former analysis conducted with only tomato-ToCMoV samples (Tables 3 and 5). *TIP41* remained as the most stable gene and *EXP* moved from the third to the second place. Surprisingly, *EF1* gene, previously ranked as the second most stable gene, was ranked as the least stable gene in this new broader analysis. However, when the four pathosystems were taken into account, *EF1* and *GAPDH* were classified as the least stable genes rather than *TUB* and *ACT*.

Overall, nearly all reference genes had a suitable performance and showed very high stability measurements in all experimental conditions tested. To decide the best genes to be used in our forthcoming qPCR gene expression analysis, we proceeded a comprehensive ranking by ordering the ten candidate genes according to their stability classification given in each program and then classified them by a simple ranking average. This strategy to evaluate individual gene stability taking into account the outputs from the three algorithms proved to be effective, as previously suggested [28]. Therefore, the combined analysis of the ranking order was able to deliver a common single list of stable genes, which will be effectively used in our experimental conditions. *TIP41* gene was identified as the most stable gene considering the both entire datasets here analyzed. Likewise, *TIP41* gene, coding for a TIP41-like family protein, was also recommended as internal reference gene in a consensus ranking of reference genes during tomato development [11] and also in *Arabidopsis* [39] and *Brassica napus* vegetative tissues [40].

Our analysis also pointed *EF1* as the second most stable gene for tomato-ToCMoV pathosystem and *EXP* for the four pathosystems together. *EF1* gene, coding for an elongation factor 1α, is a well-known reference gene and was previously tested as a candidate for internal control during the early stages of the tomato infection by PepYMV [14]. Interestingly, in the aforementioned report, *APT1* displayed the smallest variation among all treatments whereas in our study with ToCMoV the same gene was one of the more variable and ranked at eighth position (Table 3). *EF1* gene was also identified as the most stable among eight reference genes evaluated in tomato under growth conditions of nitrogen starvation and low temperature [13]. On the other hand, *EXP* is a less conventional internal control gene with unknown function but was also identified as stably expressed in *Arabidopsis* [39]. Despite the frequent use of *TUB* gene as internal control in qPCR gene expression studies involving plant-begomovirus interactions [35–37], our analyses pointed *TUB* gene, together with *ACT*, as the least stable in tomato-ToCMoV pathosystem. In accordance with our results, recent studies of reference genes evaluation, identified *TUB* gene as the least stable in citrus plants inoculated with CiLV-C [27] and in both *Arabidopsis* and tomato seeds [12]. In the same study, the *ACT*, another “classical” used plant reference gene, even been validated as stably expressed in tomato seeds, is surprisingly one of the most unstable gene in *Arabidopsis* seeds [12]. However, the stability ranking of these genes changed when the five pathosystems were taken into account, These observations show that even traditional plant reference genes, as *TUB*, *ACT* and *GAPDH*, have considerable variation in expression under our experimental conditions and corroborate the consensus that several reference genes must be evaluated for each given experimental condition [10].

To validate the reliability of the selected reference genes, the expression of two target genes (*NECRO* and *HR-Li*), both associated with cell death and defense response in plants [29, 30], was evaluated. As expected, the gene expression behavior and the magnitude of relative expression change depended on pathosystems studied and the reference gene combination used. These results clearly demonstrate that an inadequate choice of reference genes could lead to an erroneous analysis and interpretation of relative expression.

## Conclusions

Taking into account the results presented here, we have selected *TIP41* and *EF1* to be used in combination as reference genes in the forthcoming expression qPCR analysis of target genes for ‘Santa Clara’- and ‘LAM 157’-ToCMoV interaction studies. Pairwise variation analysis indicated that only two genes would be necessary for an optimal normalization of qPCR data and the addition of the third reference gene would not improve the analysis reliability. Likewise, *TIP41* and *EXP* reference genes proven to be stably expressed in broader experimental conditions, involving the interaction of five tomato genotypes (‘Santa Clara’; ‘Moneymaker’; “LAM 157’; ‘LAM 144S’ and ‘LAM 144R’) with four virus combinations (ToCMoV, GRSV, ToCV, and ToBMV+TMV). These conditions also considered differently aged leaves (from 3 to 100 DAI) and three inoculation methods (biolistics; whitefly and mechanical transmissions). The usefulness of *TIP41* and *EF1*or *EXP* as suitable reference genes was further validated as normalizers in relative expression of target genes. This is the first systematic identification and validation of reliable reference genes for these four tomato-virus pathosystems. The results will benefit and help to improve the accuracy of gene expression analysis in large experimental conditions of tomato-virus interaction studies. In addition, we recommended that a preliminary careful evaluation of reference genes stability should be performed, including several “classical” and less conventional candidate genes and using at least three statistical algorithms, in order to avoid inaccurate qPCR data normalization for each new experimental setup.

## Supporting Information

S1 Fig Ranking of reference genes by geNorm, NormFinder, and BestKeeper. Ranking of candidate reference genes based on their expression stability values estimated by geNorm, NormFinder, and BestKeeper algorithms. Analysis conducted with the entire dataset and individual (genotype, treatments or time) subsets in ‘LAM 157’ and ‘Santa Clara’ interaction with ToCMoV.
(XLSX)
